# Thromboembolic events in polycythemia vera

**DOI:** 10.1007/s00277-019-03625-x

**Published:** 2019-03-08

**Authors:** Martin Griesshammer, Jean-Jacques Kiladjian, Carlos Besses

**Affiliations:** 1 University Clinic for Hematology, Oncology, Hemostaseology and Palliative Care, Johannes Wesling Medical Center Minden, UKRUB, University of Bochum, Hans-Nolte-Straße 1, 32429 Minden, Germany; 2 Hôpital Saint-Louis, AP-HP, Centre d’Investigations Cliniques (CIC 1427), Université Paris Diderot, INSERM UMRS 1131, 1 Avenue Claude Vellefaux, Paris, France; 3 Hospital del Mar-IMIM, Passeig Marítim 25-29, 08003 Barcelona, Spain

**Keywords:** Polycythemia vera, Thromboembolic events, Thrombosis, Interferon, JAK inhibitors

## Abstract

Thromboembolic events and cardiovascular disease are the most prevalent complications in patients with polycythemia vera (PV) compared with other myeloproliferative disorders and are the major cause of morbidity and mortality in this population. Moreover, a vascular complication such as arterial or venous thrombosis often leads to the diagnosis of PV. The highest rates of thrombosis typically occur shortly before or at diagnosis and decrease over time, probably due to the effects of treatment. Important risk factors include age (≥ 60 years old) and a history of thrombosis; elevated hematocrit and leukocytosis are also associated with an increased risk of thrombosis. The goal of therapy is to reduce the risk of thrombosis by controlling hematocrit to < 45%, a target associated with reduced rates of cardiovascular death and major thrombosis. Low-risk patients (< 60 years old with no history of thrombosis) are managed with phlebotomy and low-dose aspirin, whereas high-risk patients (≥ 60 years old and/or with a history of thrombosis) should be treated with cytoreductive agents. Interferon and ruxolitinib are considered second-line therapies for patients who are intolerant of or have an inadequate response to hydroxyurea, which is typically used as first-line therapy. In this review, we discuss factors associated with thrombosis and recent data on current treatments, including anticoagulation, highlighting the need for more controlled studies to determine the most effective cytoreductive therapies for reducing the risk of thrombosis in patients with PV.

## Introduction

Thromboembolic events (TEs) are a major complication of myeloproliferative neoplasms (MPNs) [1]. Patients with MPNs have an increased risk of thrombotic events compared with the general population, with these events adding to the morbidity and mortality associated with MPNs [2, 3]. TEs and cardiovascular disease are more prevalent in polycythemia vera (PV) than in other myeloproliferative disorders [2–4]. A retrospective analysis of patients with MPNs from the Swedish Cancer Registry (*n* = 9429; PV, *n* = 3001), including patient data from 1987 to 2009 with follow-up until 2010, reported that at 3 months after diagnosis, patients with PV had an approximately 3- and 13-fold higher risk of arterial thrombosis and venous thrombosis, respectively, compared with controls matched for age and sex [1]. A recent single-center study of 526 patients with MPNs with an overall study period of 3497.4 years reported an incidence rate of 1.7% venous TEs per patient/year [5]. Overall, 38.4% of all venous TEs occurred before or at diagnosis of the MPNs, with 55.6% occurring at uncommon sites, such as splanchnic or cerebral veins. Venous TEs were significantly more common in women (*P* = .028), patients positive for a Janus kinase 2 (*JAK2*) mutation (*P* = .018), and those diagnosed with PV (*P* = .009).

A vascular complication may lead to the diagnosis of PV [6], with thrombosis (arterial or venous) being the most frequent clinical complication [3]. Thrombosis and TEs are observed in approximately 39–41% of patients with PV [6, 7], and arterial and venous thromboses are the main causes of morbidity and mortality in these patients [8]. Arterial thromboses comprise 60–70% of all cardiovascular events in patients with PV and include transient ischemic attack (TIA), stroke, acute myocardial infarction, and peripheral arterial occlusion [8]. Venous thromboses occur as deep vein thromboses of the extremities, pulmonary emboli, and venous thromboses in unusual sites such as splanchnic or sinus sagittalis superior vein thromboses. In recent observational studies, acute coronary syndrome, stroke, cerebrovascular arterial thrombosis, and acute myocardial infarction were among the most common arterial events in patients with PV, whereas deep vein thrombosis, splanchnic vein thrombosis, pulmonary embolism, and superficial venous thrombosis were among the most common venous events [9, 10].

Thrombotic events in patients with PV often occur years before diagnosis of the disease, with thrombosis before diagnosis occurring in 12–15% of patients [6, 10, 11], and occurring more frequently shortly before diagnosis [3, 6]. In the study by the Gruppo Italiano Studio Policitemia, in which 1213 patients were followed-up for 20 years, most thrombotic events (64%) occurred shortly before or at diagnosis, with most events occurring in the 2 years preceding diagnosis [6]. Similar findings were seen in the European Collaboration on Low-Dose Aspirin in Polycythemia Vera (ECLAP) study [3] and in the real-world analysis of the MPN registry of the Study Alliance Leukemia, in which two-thirds of all events occurred shortly before or at the time of diagnosis [9]. In general, arterial thrombotic events were more common than venous thrombotic events before or at diagnosis, with approximately 16–27% of patients reporting arterial events and 7–12% reporting venous events before or at diagnosis [3, 12–14]. In a single-center study of 587 patients with PV, acute coronary syndrome was the most common arterial event (45%) that occurred before or at the time of diagnosis, whereas splanchnic vein thrombosis (45%) was the most common venous event [11]. Similarly, the rates of thrombosis after diagnosis are highest shortly after diagnosis and decrease over time [3, 6]. The study by the Gruppo Italiano Studio Policitemia reported that the incidence of thrombosis after diagnosis was 3.4% per year in 1995 [6], but more recent analyses from the CYTO-PV Group (2013) and the International Working Group for Myelofibrosis Research and Treatment (IWG-MRT; 2014) reported rates of 2.7% [13] and 2.6% [12], respectively. Decreases over the last 2 decades in the rate of thrombosis after diagnosis are likely due to advances in treatment options and more aggressive management of cardiovascular risk factors [1, 12].

As mentioned, TEs are associated with an increased risk of mortality in patients with PV. In the ECLAP study (*n* = 1638), cardiovascular mortality accounted for 45% of all deaths (mean follow-up, 2.7 years), mainly due to coronary heart disease (15%), congestive heart failure (8%), and nonhemorrhagic stroke (8%) [3]. Since TEs have such a substantial impact on the clinical course of patients with PV, this review discusses current treatment options for patients with PV that may help mitigate the risk of TEs.

## Risk factors for TEs in PV

### Clinical factors

Age and a history of thrombosis have been identified as the most important clinical risk factors for thrombosis in patients with PV [3, 15]. Age > 65 years (relative risk, 2.08 [95% CI, 1.25–3.45]) and a history of thrombotic events (relative risk, 2.09 [95% CI, 1.55–2.81]) were the 2 most significant prognostic indicators of cardiovascular events in the ECLAP study [3], with age > 65 years identified as the most important risk factor for major thrombosis (hazard ratio [HR], 2.89 [95% CI, 1.98–4.22]) [16]. Furthermore, the risk of cardiovascular events increased with age [3]. Recently, age ≥ 65 years was included by the British Society for Haematology as a defining clinical feature of high-risk PV, further emphasizing its prognostic importance [17].

A history of thrombotic events is highly predictive of new thrombotic events [3]. In the ECLAP study, prior venous thrombosis was significantly associated with subsequent venous thrombotic events (HR, 4.19 [95% CI, 2.01–8.72), and prior arterial thrombosis was significantly associated with subsequent arterial events (HR, 2.07 [95% CI, 1.40–3.06]) [16]. More recent studies have confirmed these observations and further suggest that arterial and venous events have distinct risk factors [11, 12, 14]. For instance, patient sex may influence whether arterial or venous thrombotic events occur, with arterial thrombosis more commonly reported in men than in women (18% vs 14%; *P* = .02) and venous thrombosis more frequently reported in women than in men (9.3% vs 5.4%; *P* < .01) [14]. A study by the IWG-MRT of 1545 patients with PV found that prior arterial events, as well as hypertension, were predictors of subsequent arterial thrombosis; prior venous events and age ≥ 65 years predicted venous thrombosis [12]. The study by Cerquozzi and colleagues found that prior arterial events and hyperlipidemia were predictive of subsequent arterial events, whereas prior venous events, major hemorrhage at diagnosis, and leukocytosis (white blood cell [WBC] count of ≥ 11 × 10^9^/L) predicted venous events [11]. Cerquozzi et al. also explored the association of cardiovascular risk factors with the occurrence of arterial or venous events at or following diagnosis and found that older age (≥ 60 years), hypertension, diabetes, hyperlipidemia, and normal karyotype were associated with arterial events, whereas younger age (< 60 years), female sex, palpable splenomegaly, and history of major hemorrhage were associated with venous events. Of note, leukocytosis (WBC count of ≥ 11 × 10^9^/L) was associated with overall thrombosis.

Recently, a retrospective study of 604 patients with low-risk PV reported that younger age (50–60 years) and arterial hypertension were risk factors for developing arterial thrombotic events; however, these risk factors were not associated with an increased rate of venous events [18]. The association between younger age and arterial thrombosis may be specific to patients with low-risk PV because most other analyses have reported that older age is associated with arterial thrombosis [11, 16].

### Hematologic parameters

Hematologic conditions of concern in this population include erythrocytosis, leukocytosis, and thrombocytosis. Firstly, elevated hematocrit (HCT) resulting from excessive erythrocytosis can increase blood viscosity, reduce blood return through the venous system, and increase platelet adhesion [19–21]. Increased blood viscosity promotes blood clot formation [22] and increased platelet activation at the vessel wall [21]. The Tromsø study demonstrated that elevated HCT was significantly associated with an increased risk of venous thromboembolism in the general population (5% increase in HCT; HR, 1.35 [95% CI, 1.17–1.55]) [20]. A similar association was observed early on in patients with PV [6, 23, 24], and a small retrospective study found that the incidence of thrombosis increased linearly in patients with an HCT that was > 45% (range, 46–52%) [23].

The CYTO-PV study (NCT01645124), a prospective, randomized, clinical study (*n* = 365), demonstrated that patients who maintained a target HCT of < 45% had a lower rate of cardiovascular deaths and major thrombotic events than those with a target HCT of 45–50% [13]. The results showed that the incidence of death from cardiovascular events or major thrombosis was 1.1 per 100 person-years in the group maintaining a target HCT of < 45% and 4.4 per 100 person-years in the high-HCT group. In addition, cardiovascular events occurred in 4.4% of patients who achieved a target HCT of < 45% compared with 10.9% of patients with HCT between 45 and 50% (HR, 2.69 [95% CI, 1.19–6.12]; *P* = .02) [13]. These findings support the treatment recommendations set forth by the European LeukemiaNet (ELN) and IWG-MRT, as well as the European Society for Medical Oncology Clinical Practice Guidelines, and show that maintaining a target HCT of < 45% should be an important treatment goal in the management of patients with PV [15, 25].

In the case of leukocytosis, several studies have identified an association between leukocytosis and an increased risk of thrombosis in patients with PV [16, 26–29]. Leukocytosis was first reported as an independent risk factor for arterial thrombosis in an analysis of the ECLAP study [16], in which patients with a WBC count of > 15 × 10^9^/L had a significant increase in the risk of arterial thrombosis, particularly myocardial infarction, compared with patients with a WBC count of < 10 × 10^9^/L (*P* = .017). Several other studies have also reported an association between leukocytosis and thrombosis [26–29]. In one study, leukocytosis was found to be predictive for venous thrombosis during follow-up (WBC count > 15 × 10^9^/L; *P* = .005) [26]. Another study found that leukocytosis was an independent predictor of arterial recurrence (WBC count > 12.4 × 10^9^/L; HR, 3.35 [95% CI, 0.40–20.53]); an increased leukocyte count was also correlated with the occurrence of myocardial infarction [27] and found to be prognostic for reduced survival [14]. A subanalysis of the randomized CYTO-PV study supported previous studies and indicated that an increase in the risk of thrombosis was evident in patients with a WBC count of > 7 × 10^9^/L; the risk of thrombosis was significantly increased in patients with a WBC count of > 11 × 10^9^/L (*P* = .02) [28]. In the updated ELN recommendations, a leukocyte count of > 15 × 10^9^/L is considered an indication to start cytoreductive therapy [15].

Lastly, the association between thrombocytosis and thrombosis is not clear in PV [30]. Patients with PV have an increase in thromboxane synthesis, suggesting that platelet activation is a contributor to the increased risk of thrombosis in these patients [4]. Findings from the ECLAP study further support this observation. In this study, patients who received aspirin, which targets thromboxane-dependent platelet activation, had a reduced rate of any thrombosis (HR, 0.42 [95% CI, 0.24–0.74]; *P* = .003), suggesting that platelet activation, but not necessarily thrombocytosis, contributes to thrombosis in patients with PV. In general, no clear relationship between platelets and thrombosis has been established [30–32], and, in some cases, high platelet count correlates more closely with a higher risk of bleeding than with an increased rate of thrombosis [33, 34]. However, a lack of sustained response in platelet counts (< 400 × 10^9^/L) in patients with PV treated with hydroxyurea was associated with higher rates of thrombosis (*P* = .04) and bleeding (*P* = .009) in a retrospective study of Spanish patients with PV [35]. In fact, that study is one of the few pointing to thrombocytosis as a risk factor for thrombosis but found that bleeding was a more substantial problem.

### Inflammation

The level of C-reactive protein (CRP), a marker of inflammation, is elevated in patients with PV and may also be associated with an increased risk of TEs. In a population-based study, CRP level was associated with the occurrence of thrombotic events, including myocardial infarction, stroke, and venous thrombosis [36]. In a study by Barbui and colleagues [37], higher rates of major thrombosis were associated with increasing CRP levels (*P* = .001), with the highest level of CRP doubling the risk of thrombosis. Higher CRP level also correlated significantly with a *JAK2* V617F allele burden of > 50% (*P* = .003) [37].

### Molecular risk factors (*JAK2* V617F mutation)

The association between *JAK2* allele burden and thrombotic risk is uncertain; however, recent studies have shown that patients with MPNs who carry the *JAK2* V617F mutation have an increased risk of thrombotic complication [30]. A prospective study in 173 patients with PV was conducted to determine the association between *JAK2* V617F allele burden and clinical outcomes [38]. A high *JAK2* V617F allele burden (> 75%) was associated with a 3.56-fold higher relative risk (95% CI, 1.47–7.1; *P* = .004) of total thrombosis compared with a reference population. Risk factors associated with thrombosis included age (*P* = .027), previous thrombosis (*P* = .041), leukocytosis (*P* = .047), and *JAK2* V617F allele burden (*P* = .014). In addition, the presence of the *JAK2* V617F mutation in the red cell compartment and potentially in endothelial cells may induce the expression of abnormal proinflammatory and proadherent phenotypes that may further increase the risk of thrombosis [39, 40].

## Preventing thromboembolic events: treatment options in PV

Therapy for PV aims to reduce the risk of thrombosis and bleeding, to control symptoms, to delay transformation to myelofibrosis (MF) or acute leukemia/myelodysplastic syndromes (MDS), and to manage special situations [3, 41]. Given the high mortality associated with thrombotic events in patients with PV, the first goal of therapy is to reduce the risk of thrombosis, mainly by controlling HCT to < 45% [15], a target associated with reduced rates of cardiovascular death and major thrombosis [13]. Therapy for the treatment of PV is dependent on the patient’s thrombotic risk, which is currently based on age and history of thrombosis [15, 30, 42]. Patients < 60 years old with no history of thrombosis are categorized as low risk, whereas those ≥ 60 years old and/or those with a history of thrombosis are considered high risk [15]. Current guidelines recommend managing low-risk patients with phlebotomy and low-dose aspirin, whereas high-risk patients should be treated with cytoreductive agents, with hydroxyurea and recombinant interferon alfa as first-line therapies and interferon and ruxolitinib as second-line therapies in patients who are intolerant of or have inadequate response to hydroxyurea [15].

However, findings from a recent retrospective study by Barbui and colleagues suggest that there may be a role for cytoreductive therapy in the primary prevention of TEs in some patients with low-risk PV [18]. In this study, 604 patients with low-risk PV were treated with aspirin and phlebotomy (median duration, 4.9 years) to keep the target HCT < 45%; however, 12% of patients experienced 84 major thrombotic events (venous, 45%; arterial, 55%). Arterial hypertension was significantly associated with a higher rate of arterial events in these patients, suggesting that patients with low-risk PV with arterial hypertension may require more intensive therapy, including cytoreductive therapy and/or antihypertensive treatments, such as angiotensin-converting-enzyme inhibitors [18]. However, prospective studies are needed to assess the most appropriate therapy.

In addition to cytoreduction, antiplatelet agents are generally used to treat patients with a history of arterial thrombosis, and those with a history of venous events are treated with anticoagulants (e.g., vitamin K antagonists [VKAs]) [43]. Findings from a recent study showed the benefits associated with the use of cytoreductive therapy in combination with antithrombotic drugs in patients with a history of TEs. This study of 597 patients with MPNs (PV, *n* = 184) examined the benefit-risk profile of cytoreductive drugs along with antiplatelet and antithrombotic therapies that were started after an initial TIA (*n* = 270; PV, *n* = 77) or ischemic stroke (*n* = 327; PV, *n* = 107) [42]. Treatment included antithrombotic therapy (aspirin, 85% of patients) and cytoreductive drugs (hydroxyurea, 78% of patients). The composite incidence of recurrent TIA and ischemic stroke, acute myocardial infarction, and cardiovascular death was 4.2% and 19.2% at 1 and 5 years after the index event, respectively, which was lower than that in the general population. Cytoreductive therapy was a strong protective factor (HR, 0.24), and the rate of major bleeding was similar to that in the general population (0.90 per 100 patient-years), suggesting an advantageous benefit-risk profile of cytoreductive and antithrombotic therapy [42].

Similarly, cytoreduction in combination with oral anticoagulants may also help prevent the recurrence of thrombosis, especially venous thrombosis, in patients with PV [5, 43–45], with one study reporting a 2.8-fold reduction in the risk of thrombotic recurrence with VKA treatment [43]. In a retrospective study that examined the rate of recurrence of arterial and venous thrombosis in 494 patients (PV, *n* = 235; essential thrombocythemia [ET], *n* = 259) with previous arterial (67.6%) or venous (31%) thrombosis, cytoreduction was the only treatment significantly associated with a reduction in the risk of recurrence (multivariable HR, 0.53 [95% CI, 0.38–0.73]; *P* = .0002) [44]. However, patients treated with oral anticoagulants plus cytoreduction had the lowest rate of recurrences (17.8%) compared with those treated with cytoreduction (50.0%), antiplatelet agents (35.2%), or anticoagulation alone (44.1%). When stratified by type of first event (i.e., arterial vs venous), cytoreductive treatment was associated with a significant decrease in recurrence of arterial thrombosis (HR, 0.47 [95% CI, 0.31–0.70]; *P* = .0003), whereas anticoagulants (HR, 0.32 [95% CI, 0.15–0.64]; *P* = .001) or antiplatelet therapies (HR, 0.42 [95% CI, 0.22–0.77]; *P* = .006) were associated with a significant decrease in the risk of recurrent venous thrombosis [44]. A study by De Stefano and colleagues (*n* = 206; PV, 46.6%) reported similar findings, with a lower incidence rate of recurrent venous thrombosis per 100 patient-years observed in patients receiving VKAs (4.7 [95% CI, 2.8–7.3] vs 8.9 [95% CI, 5.7–13.2]; *P* = .03) [45]. Duration of treatment was also assessed, with findings suggesting that long-term treatment may lead to lower incidence rates of recurrence per 100 patient-years compared with stopping VKA treatment (5.3 [95% CI, 3.2–8.4] vs 12.8 [95% CI, 7.3–20.7]; *P* = .008) [45]. The benefits of prolonged treatment with anticoagulants in patients with MPNs were also observed in the study by Wille et al. [5]. In this study, recurrent venous TEs were observed in 36.1% of patients who terminated prophylactic anticoagulation and in only 8.6% of patients who continued anticoagulation therapy (*P* = .0127). Most patients with recurrent venous TEs (81.3%) were not receiving anticoagulants at the time of recurrence. Given that bleeding complications are a major concern among patients taking anticoagulation, physicians may recommend shortening the duration of treatment with anticoagulants. However, in these studies, treatment with anticoagulants did not significantly increase the incidence of major bleeding, supporting long-term use of anticoagulants such as VKAs in patients with MPNs who have a history of thrombotic events [5, 43–45].

### Aspirin and phlebotomy

Phlebotomy is one of the recommended first-line treatments for patients with PV [13, 15]. Phlebotomy helps control HCT, with the goal of maintaining HCT to < 45% [13]. However, a study evaluating the need for additional phlebotomies in 533 patients with PV who were receiving hydroxyurea treatment showed that a higher intensity of treatment with phlebotomy was related to an increased risk of thrombotic events: patients requiring ≥ 3 phlebotomies per year had a higher risk of thrombosis compared with patients needing ≤ 2 phlebotomies per year (20.5% vs 5.3% at 3 years; *P* < .0001) [46]. However, a recent analysis of the ECLAP and CYTO-PV studies suggested that there is no correlation between the intensity of the phlebotomy regimen and the risk of thrombosis in patients with PV [47].

The ECLAP study demonstrated that treatment with aspirin prevented thrombotic complications in patients with PV [4]. Low-dose aspirin reduced the risk of nonfatal myocardial infarction, nonfatal stroke, pulmonary embolism, major venous thrombosis, and death from cardiovascular causes (HR, 0.40 [95% CI, 0.18–0.91]; *P* = .03). Consistent with these findings, in the ECLAP study, antiplatelet therapy was significantly associated with a lower risk of cardiovascular events (HR, 0.72 [95% CI, 0.53–0.97]; *P* = .0315) [3].

### Hydroxyurea

Hydroxyurea is the most commonly used first-line cytoreductive therapy in patients with PV [15]. This practice is based mainly on studies conducted by the Polycythemia Vera Study Group (PVSG) and the French Polycythemia Study Group [41, 48, 49]. The PVSG study was conducted in 51 patients with PV who were all treated with hydroxyurea, and its efficacy was compared retrospectively with that in 194 patients treated with phlebotomy only [50]. Hydroxyurea treatment led to a reduction in the number of thrombotic events (9.8% vs 32.8% in the phlebotomy group; *P* = .009). The French Polycythemia Study Group compared hydroxyurea therapy with pipobroman therapy in a randomized study of 292 patients with PV who were < 65 years old, with a median follow-up of 9 years [48]. Initially, each therapy led to a complete hematologic remission in all but 5 patients (pipobroman, *n* = 3; hydroxyurea, *n* = 2). In the long-term analyses of this study, no significant differences in the incidence of thrombosis were seen between the 2 therapies, but the risk of leukemic transformation was clearly higher in the pipobroman arm. The final results of this trial showed that, with a median follow-up of 16 years, pipobroman presented a very high risk of evolution to acute leukemia/MDS (cumulative incidence of 52% at 20 years vs 24% with hydroxyurea) and that evolution to acute leukemia/MDS was the most common cause of death in this cohort of patients [41].

More recently, Barbui and colleagues examined 1042 patients included in the ECLAP study, during the follow-up phase (median, 2.8 years), who received phlebotomy only (*n* = 342) or hydroxyurea only (*n* = 681) to maintain an HCT of < 45% [51]. A lower incidence of fatal and nonfatal cardiovascular events was reported in the hydroxyurea group than in the phlebotomy group (3.0 vs 5.8 per 100 patient-years, respectively; *P* = .002) [51]. In addition, in the high-risk group (> 60 years and/or prior history of thrombosis), treatment with hydroxyurea was associated with a significantly lower rate of fatal and nonfatal cardiovascular events (4.8 vs 8.7 per 100 patient-years), hematologic transformations (0.1 vs 1.5 per 100 patient-years), and overall mortality (0.1 vs 0.5 per 100 patient-years) compared with phlebotomy alone [51]. However, as mentioned previously, cytoreductive therapy alone may not be sufficient to prevent recurrent thrombosis. In the study by Wille and colleagues, only 25% of recurrences of venous TEs occurred when patients were not receiving cytoreductive treatment [5]. Interestingly, hematologic parameters were controlled, suggesting that the addition of anticoagulation therapy to cytoreduction is important in preventing venous TEs. Importantly, no significant increase in major bleeding was observed in patients who received concomitant anticoagulation and cytoreduction.

Although hydroxyurea treatment lowers the rate of cardiovascular events, approximately 15–24% of patients may eventually become resistant to or experience unacceptable adverse effects from this treatment (hydroxyurea intolerance) [35, 52]. Resistance is important to recognize since it is associated with higher risk of death and transformation. The ELN has published criteria for identifying patients experiencing clinical resistance to or intolerance of hydroxyurea [53]. Cytopenias, uncontrolled myeloproliferation, and increased phlebotomy requirements are associated with hydroxyurea resistance, and skin toxicity, mucocutaneous toxicity, gastrointestinal toxicity, and fever are associated with hydroxyurea intolerance [35].

Skin toxicity, one of the more common adverse events associated with hydroxyurea treatment, has been reported in approximately 5–11% of patients with MPNs [54–56]. In a retrospective study evaluating severe mucocutaneous toxicity associated with hydroxyurea in 614 patients with MPNs (PV, 34.9%), 51 patients (8.3%) reported skin toxicity after a median treatment period of 32.1 months [55]. In patients with PV, 35.3% reported experiencing skin toxicity; however, a similar proportion did not (34.8%; *P* = .53). Permanent discontinuation of hydroxyurea was reported in 27 patients (52.9%) overall [55]. In a large retrospective study of 3411 patients with MPN (PV, *n* = 963), 536 patients were treated with hydroxyurea and evaluated for drug-related toxicities [56]. Hydroxyurea-related toxicities were reported in 184 patients (5%; PV, *n* = 61 [33%]), which included mucocutaneous lesions (*n* = 167 [90.8%]; PV, *n* = 57 [94%]) [56]. The overall discontinuation rate due to hydroxyurea toxicity was 5%. This is lower than discontinuation rates previously reported, including rates observed in the UK Medical Research Primary Thrombocythemia 1 study in high-risk ET (10.6%) [54]. However, gastrointestinal toxicities were not reported in the retrospective study but were reported in the Primary Thrombocythemia 1 study, which may have contributed to the difference in discontinuation rates. More recent prospective, albeit smaller, studies suggest that rates of hydroxyurea-related skin toxicity in patients with MPNs may be higher. A prospective, noninterventional study conducted in Germany found that 43% of patients with MPNs (PV, *n* = 55; ET, *n* = 55; MF, *n* = 41) exposed to hydroxyurea (median exposure, 46 months) presented with skin abnormalities compared with 7% of patients treated with other therapies (ruxolitinib, anagrelide, or pegylated interferon alfa; *P* = .0001) [57]. Overall, 13% of patients discontinued due to skin toxicity vs 2% of patients who were not treated with hydroxyurea (*P* = .014). Another prospective, single-center study assessed the incidence of cutaneous adverse events in patients with ET (*n* = 74) or PV (*n* = 36) treated with hydroxyurea and reported that, overall, 60% of patients (66 of 110) experienced a cutaneous adverse event, with 54% of those patients (36 of 66) developing a serious cutaneous adverse event [58]. At 48 months, the cumulative incidence was 70% for any cutaneous adverse event and 20% for any serious cutaneous adverse event. Overall, adverse events and discontinuation rates due to hydroxyurea therapy were relatively low in retrospective studies but were more frequently reported when prospectively tracked; therefore, physicians need to be aware that skin toxicities with hydroxyurea may be more frequent than expected and can be severe [54, 56] and that dermatologic monitoring is recommended in these patients, especially in those who present with actinic keratoses or a history of squamous cancer before the initiation of hydroxyurea.

### Interferon

Interferon has been shown to induce high rates of hematologic and molecular responses in patients with PV [59, 60] and is recommended as frontline therapy, especially for young patients who need long-term treatment, and as second-line therapy for patients with PV who are intolerant of or have inadequate response to hydroxyurea [15, 61]. Interferon has been evaluated in several small studies, including some phase 2 studies, in which it has been shown to be effective in achieving hematologic remission, reducing *JAK2* V617F allele burden, and reducing rates of thrombosis [62–64]. Discontinuation occurs in approximately 25% of patients, and tolerability is improved with the use of low doses at initiation. In some patients, interferon may achieve sustained hematologic and molecular responses even after discontinuation of therapy.

PROUD-PV (NCT01949805), a randomized, controlled, multicenter, phase 3 trial comparing the efficacy, safety, and tolerability of hydroxyurea and ropeginterferon alfa-2b in 257 patients with PV who were not resistant to or intolerant of hydroxyurea showed noninferiority of ropeginterferon alfa-2b compared with hydroxyurea in terms of complete hematologic response according to ELN criteria, with spleen normality at 12 months [65, 66]. Forty-five percent of patients had a hematologic response, with mean HCT decreasing from 48 to 42%, leukocyte counts decreasing from 12 to 6 × 10^9^/L, and platelet counts decreasing from 530 to 260 × 10^9^/L. The need for phlebotomy within 3 months decreased from 86 to 6%. A *JAK2* molecular response was achieved in 37% of patients, with mean mutant *JAK2* allele burden decreasing from 42.5 to 28.7%. However, observed spleen reductions with ropeginterferon were not clinically relevant due to the almost-normal baseline spleen size in the majority of patients. Overall, ropeginterferon alfa-2b had a better adverse event profile compared with hydroxyurea and was well tolerated. Although more patients in the ropeginterferon alfa-2b group experienced cardiovascular events (3.1%, including cardiac failure, thrombotic event, and stroke), endocrine events (3.1%, including autoimmune thyroiditis and hypo- or hyperthyroidism), or psychiatric events (1.6%, including anxiety, depression, and mood altered), the latter being a well-known toxicity of interferon, the incidence of these events was not statistically significant compared with that in the hydroxyurea group. A 12-month continuation of this study (CONTINUATION-PV; NCT02218047) comparing ropeginterferon alfa-2b with best available therapy (BAT) showed that, after 24 months of treatment, complete hematologic response (CHR) rates were higher in the ropeginterferon alfa-2b group compared with the BAT group (CHR, 70.5% vs 49.3%, respectively; *P* = .01); however, cardiovascular and vascular disorders occurred at a rate of 10.2% in the ropeginterferon alfa-2b group and 5.5% in the BAT group. Overall, treatment-related adverse events were reported in 70% and 77% of patients treated with ropeginterferon alfa-2b and BAT, respectively [67].

### Ruxolitinib

Ruxolitinib is the only JAK inhibitor approved for the treatment of patients with PV, specifically those who are resistant to or intolerant of hydroxyurea [15, 68]. Ruxolitinib was evaluated in 2 phase 3 studies in patients who were resistant to or intolerant of hydroxyurea and had splenomegaly (RESPONSE; NCT01243944 [69]) or no palpable spleen (RESPONSE-2; NCT02038036 [70]). Both studies met their primary endpoints and showed that ruxolitinib was superior to BAT in providing HCT control without phlebotomies and improving symptom burden in this patient population, regardless of spleen size. In the 208-week (4-year) analysis of the RESPONSE study, 37% of patients were still receiving treatment with ruxolitinib vs no patients in the BAT arm [71].

Although the RESPONSE and RESPONSE-2 studies were not powered to assess TEs, fewer thrombotic events were seen in patients treated with ruxolitinib compared with BAT. In the RESPONSE study, thrombotic events occurred in 1 patient (0.9%) treated with ruxolitinib and 6 patients (5.4%) treated with BAT (1.8 vs 8.2 per 100 patient-years of exposure, respectively) [69, 72]. In a 4-year analysis of the RESPONSE study, the rate of TEs was lower with ruxolitinib compared with BAT (all grades, 1.2 vs 8.2 per 100 patient-years; grade 3/4, 0.7 vs 2.7 per 100 patient-years, respectively) [71]. In the primary analysis of RESPONSE-2, the corresponding rates were 1.4% (*n* = 1) with ruxolitinib and 4.0% (*n* = 3) with BAT [70]. At 80 weeks of follow-up in RESPONSE-2, embolic and thrombotic events occurred at a rate of 1.5 per 100 patient-years in the ruxolitinib group and 1.9 per 100 patient-years in the BAT group [73]. This finding may be attributed to better HCT and WBC control with ruxolitinib than with standard therapy, given that these 2 hematologic parameters have been independently linked to an increased risk of thrombotic events [13, 28]. In the primary analysis of the RESPONSE studies, the proportion of patients who achieved HCT control (i.e., ≤ 45%) was significantly higher with ruxolitinib than with BAT (RESPONSE, 60.0% vs 18.8%; RESPONSE-2, 62.0% vs 19.0%) [69, 70]. HCT control was also maintained in most patients (RESPONSE, 73% for 208 weeks; RESPONSE-2, 78% for 80 weeks) [71, 73]. Additionally, in both RESPONSE studies, the proportion of patients undergoing phlebotomy procedures was lower with ruxolitinib than with BAT. This finding could be important in assessing the risk of thrombosis given that, as described above, the intensity of treatment with phlebotomy may be related to an increased risk of thrombotic events [46].

In the RESPONSE study, ruxolitinib also led to control of WBC counts in patients with PV. A subanalysis of the RESPONSE study showed that ruxolitinib led to greater reductions in WBC counts compared with BAT or hydroxyurea. In patients with baseline WBC counts of ≥ 11 × 10^9^/L, those treated with ruxolitinib had greater mean reductions in WBC counts compared with those treated with BAT, and these reductions were maintained over time [74]. Among patients with WBC counts of > 10 or > 15 × 10^9^/L at baseline, a higher proportion of ruxolitinib-treated patients achieved an ELN response (WBC count ≤ 10 × 10^9^/L) [74]. In addition to these analyses, a meta-analysis of the COMFORT-I, COMFORT-II, and RESPONSE studies evaluated the effect of ruxolitinib on the risk of thrombosis among patients with MF or PV [75]. The rates of thrombosis were significantly lower in patients who were treated with ruxolitinib (risk ratio, 0.45 [95% CI, 0.23–0.88]). The rates of venous and arterial thrombosis also demonstrated similar risk ratios (0.46 [95% CI, 0.14–1.48] and 0.42 [95% CI, 0.18–1.01], respectively); however, these risk ratios did not reach statistical significance.

However, ruxolitinib was associated with an increased rate of herpes zoster infection compared with standard therapy (RESPONSE: exposure-adjusted rate at 4 years, 4.9 per 100 patient-years; RESPONSE-2: exposure-adjusted rate at 80 weeks, 3.8 per 100 patient-years); most herpes zoster infections were grade 1 or 2 and resolved without sequelae [71, 73]. Rates of nonmelanoma skin cancer were also increased in patients who received ruxolitinib (RESPONSE: exposure-adjusted rate at 4 years, 3.6 per 100 patient-years; RESPONSE-2: exposure-adjusted rate at 80 weeks, 0.8 per 100 patient-years for squamous cell carcinoma of skin only) [71, 73]. Prior nonmelanoma skin cancer, previous therapy (e.g., hydroxyurea) or aging may have had an impact on the nonmelanoma skin cancer rates observed with ruxolitinib. This finding was described in the 80-week follow-up data from the RESPONSE study, in which nonmelanoma skin cancers were observed in the originally randomized ruxolitinib arm, primarily in patients with a history of nonmelanoma skin cancer. However, at the 80-week analysis, exposure-adjusted rates were generally similar between the ruxolitinib and BAT arms [76]. Furthermore, all patients who developed squamous cell carcinoma of the skin in the RESPONSE-2 study at 80 weeks had prior exposure to antineoplastic therapy, including hydroxyurea [73]. It has recently been reported that there may be an increased risk of developing B cell lymphomas in patients with MF treated with ruxolitinib, in particular those presenting with a clonal immunoglobulin gene rearrangement in the bone marrow before starting ruxolitinib [77]; however, there have been no reports of B cell lymphomas in patients enrolled in the RESPONSE studies in PV. Additional studies are needed to determine the risk in this population.

### Treatment options for splanchnic vein thrombosis

MPNs are a leading cause of noncirrhotic and nonmalignant splanchnic vein thrombosis (SVT) [78]. SVT is a rare type of venous thrombosis that may involve several abdominal veins (portal, splenic, mesenteric, and hepatic) and includes Budd-Chiari syndrome, extrahepatic portal vein obstruction, and mesenteric vein thrombosis [79]. SVT is seen in all types of MPNs and is mainly observed in younger patients [78, 80, 81]. PV is the most common MPN subtype in patients with SVT [82], occurring in 0.8–2% of patients with PV [10, 12]. *JAK2* V617F is common in patients with SVT and has been detected in 96.5% of patients with SVT and MPNs and in 7% of patients with SVT who have no MPN features on bone marrow biopsy [81]. Overall, SVT has been reported to account for 7.5% of first thromboses in patients with MPNs [44].

Management of SVT in patients with MPNs may be challenging and is usually focused on preventing recurrent thrombosis, managing MPNs, and managing organ dysfunction [80]. If there are no major contraindications, anticoagulant therapy is usually recommended for all patients presenting with acute symptomatic splanchnic vein thrombosis [80, 83]. Typically, patients are started on either a full-dose low-molecular-weight or unfractionated heparin followed by VKA [80, 83]. However, the use of anticoagulant therapy should be carefully monitored given the increased risk of bleeding, which must be balanced against the need to prevent thrombosis recurrence. Patients with PV and SVT should be treated with cytoreductive therapy to maintain HCT < 45%, platelet count of ≤ 400 × 10^9^/L, and WBC count of < 10 × 10^9^/L, as proposed in current treatment guidelines [15]. However, cytoreduction has not been shown to be effective in preventing recurrence of SVT. In a retrospective study of patients with MPNs (*n* = 181), the incidence rate of recurrent events in patients treated with cytoreduction was similar to that observed in patients without cytoreductive treatment (4.2 vs 4.0 per 100 patient-years, respectively) [84]. Overall, treatment of SVT in patients with MPNs remains an unmet clinical need, and additional studies are needed to assess potential treatments.

## Conclusions

**Fig. 1:**
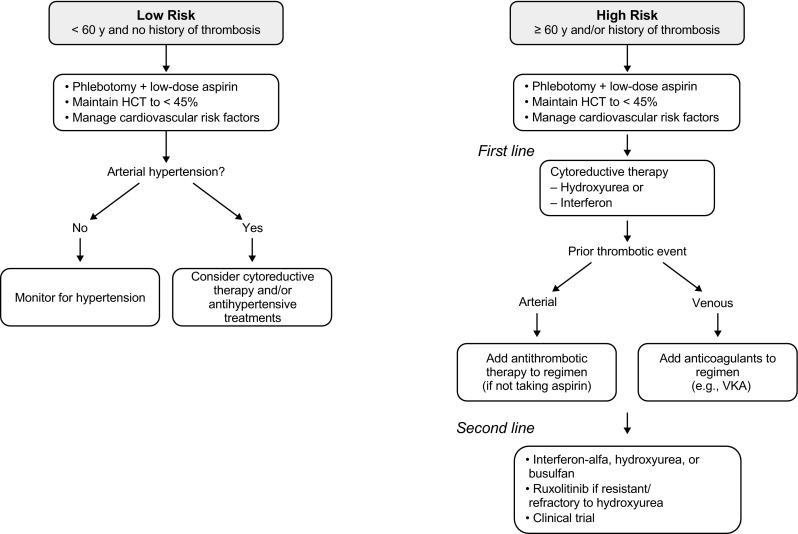
Treatment algorithm for prevention of thromboembolic events in PV. HCT, hematocrit; VKA, vitamin K antagonists

TEs and cardiovascular disease are more prevalent in PV than in other myeloproliferative disorders and represent the major cause of morbidity and mortality in these patients [2–4]. Older age and a history of thrombosis have been identified as the most important risk factors, with increased HCT and leukocytosis also being relevant risk factors for thrombosis in patients with PV [3, 15]. The goal of therapy is to reduce the risk of thrombosis by controlling HCT to < 45%, a target associated with reduced rates of cardiovascular death and major thrombosis. Patients with low-risk PV (< 60 years old with no history of thrombosis) are managed with phlebotomy and low-dose aspirin, whereas those with high-risk disease (≥ 60 years old and/or with a history of thrombosis) should be treated with a cytoreductive agent, such as hydroxyurea or interferon alfa (Fig. 1). Ruxolitinib is approved as a second-line therapy for patients who are intolerant of or have an inadequate response to hydroxyurea [15]. The use of antiplatelet therapy or VKAs, in addition to cytoreduction and phlebotomy, should also be considered to prevent secondary thromboses (Fig. 1). Interferon and ruxolitinib may be used as second-line therapies for patients who are intolerant of or have an inadequate response to hydroxyurea, especially after a TE occurring during hydroxyurea treatment [15]. However, although the use of hydroxyurea has been associated with lower incidence of cardiovascular events, additional controlled studies are needed to assess TE rates with new promising therapies, such as ropeginterferon and ruxolitinib, and to determine the most effective cytoreductive and/or combination therapies to prevent thrombosis in patients with PV. Confirmation in randomized studies of the low rate of thrombosis consistently reported in phase 2 studies of interferon alfa [61, 85] and the encouraging results observed with long-term treatment with ruxolitinib in the RESPONSE study [86] will hopefully provide new alternatives to further reduce the risk of TEs in patients with PV.
